# Overlap Syndrome in a Male Presenting With Progressive Lower Limb Weakness: A Case Report

**DOI:** 10.7759/cureus.33664

**Published:** 2023-01-11

**Authors:** Fatema M Dubai, Ahmed Isa

**Affiliations:** 1 Internal Medicine, Salmaniya Medical Complex, Manama, BHR; 2 Emergency, Shifa Al Jazeera Medical Center, Manama, BHR

**Keywords:** creatine kinase, proximal muscle weakness, membranous glomerulonephritis, sle-myositis overlap syndrome, lupus nephritis, sle, rheumatology, inflammatory myositis, systemic lupus erythematosus, overlap syndrome

## Abstract

Overlap syndrome refers to a large group of inflammatory rheumatic conditions characterized by the co-existence of clinical manifestations that include different organ systems and meet the criteria of more than one rheumatic disease. Overlap syndromes are less common compared with the conditions they encompass, for example, the global prevalence of systemic lupus erythematosus (SLE) is estimated to be 43.7 per 100,000 persons, of which only around 3.4-6.3% present with SLE-myositis overlap. Although rare, overlap syndromes commonly include lupus, rheumatoid arthritis, scleroderma, and myositis. Because overlap syndrome can manifest in several ways and has an unpredictable course, it poses a challenge to multidisciplinary teams that examine and treat patients. Therefore, we must not disregard any signs and symptoms as they might have a huge impact on the progression of the disease and the overall outcome of the treatment. We present a rare case of SLE-myositis overlap syndrome in a 44-year-old male. He initially presented with gradual weakness in the proximal muscles of the bilateral lower limb. This patient was diagnosed as having SLE with positive 5g protein/24 hours, anti-nuclear, low C3, anti-U1RNP, anti-Ro, and anti-La antibodies, as well as membranous lupus nephritis evident by the results of renal biopsy. The diagnosis of myositis was also made according to the history and evaluation of the patient, the high titer of muscle enzymes creatine kinase level, and MRI result. Although the patient tested positive for anti-U1RNP, he did not meet the criteria of mixed connective tissue disease. Eventually, the patient was found to have overlap syndrome. The prevalence of overlap between SLE and myositis is relatively rare and varies from 3.4% to 6.3%. To our knowledge, no study has discussed or reported its prevalence among males.

## Introduction

Overlap syndrome is a term used for patients who have two or more connective tissue diseases, either simultaneously or sequentially [1]. Systemic lupus erythematosus (SLE) is a multisystem autoimmune disease that has several phenotypes and can present in different clinical manifestations ranging in severity [2].

Myopathies are a group of autoimmune diseases that primarily affect muscles, usually causing weakness, along with other manifestations involving other systemic organs, most notably the heart, lungs, joints, and skin. These myopathies are further subcategorized into four main subtypes based on both the clinical phenotype and histopathology, namely, dermatomyositis, polymyositis, inclusion body myositis, and necrotizing myopathies [3]. To date, many myositis-associated and specific autoantibodies have been detected and their clinical significance on manifestation and prognosis have been elucidated [4].

While the concept of overlap syndrome might sound simple and uncomplicated at first instance; it is rare and very challenging to diagnose. Here, we present a case of SLE-myositis overlap syndrome in a 44-year-old male patient. By presenting this case we hope to highlight to clinicians the possibility of SLE-myositis overlap syndrome in male patients presenting with only one or two features of the rheumatic condition.

## Case presentation

We present the case of a 44-year-old previously healthy male not known to have any chronic medical illness. The patient was referred to our emergency department in June 2022 for electrocardiogram (ECG) changes and progressive bilateral lower limb proximal muscle weakness for the past three months. He denied having pain, tenderness, or paraesthesia over the tip of the toes. He worked as a soldier. On arrival, the patient was vitally stable. An examination revealed mild bilateral lower limb edema but was otherwise unremarkable. ECG showed sinus tachycardia with frequent premature ventricular contractions. Investigations showed high levels of creatine kinase (CK) (4,895 U/L), myoglobin (>1,000 μg/L), and urine protein (3+). Other routine laboratory parameters were within normal limits. The patient was admitted for three days in general medicine under the initial impression of rhabdomyolysis. During the hospital course, a cardiology consultation was done for the ECG changes, and the patient was managed with aggressive hydration for the high CK levels, which resulted in only a mild drop in CK levels to 4,101 U/L after three days. The patient was discharged and encouraged to have plenty of oral hydration and to minimize physical activity for a week. A renal ultrasound (USG) was booked for the patient after 10 days.

At the follow-up appointment after seven days, the patient did not notice any improvement in the weakness and started having early morning stiffness that lasted for more than 30 minutes. Further investigations were done for the patient (Table 1). Renal USG revealed slight enlargement in both kidneys, a small perirenal collection at the lower pole of the left kidney suggestive of a small peri-renal hematoma, and a small renal cyst. Other laboratory reports showed isolated proteinuria (>5 g/day), CK of 4,493 U/L, anti-nuclear antibody (ANA) speckled pattern at 1:160, low C3, anti-La positive, anti-Ro, positive and anti-U1RNP positive. In light of these findings, a referral to nephrology for further Investigations (Table 2) and management was done. Further investigations were done, which revealed the following findings: ANA positive, anti-dsDNA of 5.2 IU/mL, anti-GBM antibody positive, anti-cytomegalovirus (CMV) immunoglobulin M (IgM) negative, anti-CMV IgG positive, anti-UIRP positive, anti-streptolysin O at 12.2 mg/L, urine protein at 4,199 mg/L, and CK at 5,153 U/L.

**Table 1:** Initial serum laboratory results with reference values.

Laboratory parameter	Value	Normal range
White blood cells	5.6 × 10^9^/L	3.6–9.6 × 10^9^/L
Erythrocyte sedimentation rate	25 mm/hour	<20 mm/hour
Urea	4.9 mmol/L	3.2–8.2 mmol/L
Creatinine	31 μmol/L	53–97 μmol/L
Creatinine (urine)	5,153 μgmol/L	
Protein (urine)	4,199 mg/L	
Protein creatinine calculated ratio	814.87 mg/mmol	
24-hour urine protein	>5 g/24 hour	<0.1 g/24 hour
Estimated glomerular filtration rate	272 mL/minute/1.73m^2^	
Anti-dsDNA	2.1 IU/mL	<10
Anti-nuclear antibody screen test	Positive	
Anti-nuclear antibody (IF)	Positive speckled pattern at 1:160	
Anti-streptolysin O titer	12.2 IU/mL	0–200 IU/mL
C-reactive protein	0.74 mg/L	0–3 mg/L
Myeloperoxidase antibody	0.3 IU/mL	Negative
Proteinase 3-antineutrophil cytoplasmic antibodies	0.8	Negative
C3 level	61 mg/dL	90–180 mg/dL
C4 level	17.7 mg/dL	10–40 mg/dL
Anti-Smith antibody	Negative	Negative
Anti-La antibody	Positive	Negative
Anti-Ro antibody	Positive	Negative
Anti-U1RNP antibody	Positive	Negative

**Table 2:** Patient significant serum laboratory results (two months later) with reference values

Laboratory parameter	Value	Normal range
White blood cells	5.2 × 10^9^/L	3.6–9.6 × 10^9^/L
Erythrocyte sedimentation rate	25 mm/hour	<20 mm/hour
Urea	5.3 mmol/L	3.2–8.2 mmol/L
Creatinine	44 μgmol/L	53–97 μgmol/L
Creatinine (urine)	5,970 μgmol/L	
Protein (urine)	3,157 mg/L	
Protein creatinine calculated ratio	528.91 mg/mmol	
24-hour urine protein	5 g/24 hour	<0.1 g/24 hour
Estimated glomerular filtration rate	272 mL/minute/1.73m^2^	
Anti-dsDNA	2.1 IU/mL	<10 IU/mL
Anti-nuclear antibody screen test	Positive	
Anti-nuclear antibody (IF)	Positive speckled pattern at 1:640	
Anti-streptolysin O titer	13 IU/mL	0–200
C-reactive protein	20.7 mg/L	0–3
Rheumatoid factor	40.1	
Myeloperoxidase antibody	0.4 IU/mL	Negative
Proteinase 3-antineutrophil cytoplasmic antibodies	0.8	Negative
C3 level	127 mg/dL	90–180 mg/dL
C4 level	27.7 mg/dL	10–40 mg/dL
Anti-Smith antibody	Negative	Negative
Anti-La antibody	Positive	Negative
Anti-Ro antibody	Positive	Negative
Anti-U1RNP antibody	Positive	Negative

The patient was planned for a renal angiogram and booked for renal biopsy under the provisional diagnosis of mixed connective tissue disease with kidney involvement. He was admitted after seven days for a renal biopsy, and after 20 days was electively admitted for a renal angiogram to assess the microvascular involvement. Thus, the patient followed up with cardiology, nephrology, and rheumatology for further investigations and treatment. Echocardiography was done by the cardiologist, which was normal, and Holter showed 28% ectopic beats. The cardiologist advised the patient to start bisoprolol 1.25 mg with a follow-up. The renal biopsy showed changes consistent with membranous lupus glomerulonephritis (ISN/RPS lupus nephritis class V), activity index of 1/24, chronicity index of 3/12, and well-preserved renal parenchyma (glomerulosclerosis 0%, interstitial fibrosis and tubular atrophy <5%). The patient started following up in the rheumatology clinic and was advised MRI of the lower limb. He was started on methylprednisolone injections for three days, prednisolone 60 mg for 14 days to be tapered by 5 mg every two weeks, and mycophenolic mofetil 500 mg. The patient was presenting for weekly follow-ups in the rheumatology clinic. After eight days, he presented complaining of no improvement in his symptoms and the development of a photosensitive malar rash. The patient was advised to continue prednisolone 60 mg for one month and to increase mycophenolic acid to 1,000 mg. After 14 days, the patient came for a follow-up and started to respond to the treatment. Lower limb MRI results showed diffuse bilateral and posterior femoral muscle compartment myositis, more significantly involving the adductor and quadratus femoris muscles bilaterally. Therefore, the patient was diagnosed with SLE-myositis overlap syndrome and kept on prednisolone and mycophenolic acid with a monthly follow-up.

## Discussion

It is very difficult to differentiate myositis associated with SLE from myalgia occurring in SLE patients. SLE-myositis overlap is a very rare rheumatologic disease, with an overwhelming majority of patients presenting initially with proximal muscle weakness [5].

According to the 2019 European League Against Rheumatism/American College of Rheumatology classification, the diagnostic criteria for SLE include a positive ANA test, followed by additive weighted criteria that are grouped into seven clinical (constitutional, hematological, musculoskeletal, mucocutaneous, serosal, renal, neuropsychiatric) and three immunological (SLE-specific antibodies, anti-phospholipid antibodies, complement proteins) domains weighted from two to ten [6].

Regarding the investigations, ANA is a biomarker of SLE that can be used for screening, diagnosis, and prognosis and has a sensitivity of 95-97% but low specificity of 20%. Thus, a positive ANA does not confirm the diagnosis of SLE. Anti-dsDNA is a biomarker that indicates renal involvement and is mainly used to assess the disease activity as its level can increase during a flare-up or become undetectable during treatment. It has a 96% specificity but low sensitivity of 52-70%. Anti-Smith antibody is a biomarker associated with lupus nephritis and is used to assess disease activity in new-onset SLE due to its slow response to disease activity changes. C3 and C4 complement activation plays a significant role in SLE pathogenesis, which helps in assessing disease activity in SLE patients. Patients with simultaneously low C3 and C4 levels and a positive ANA test for an SLE diagnosis are found to have 97.6% specificity. Anti-Sjögren syndrome-related antigen A (anti-Ro) and anti-Sjögren syndrome-related antigen B (anti-La) autoantibodies are seen in up to 50% and 20% of SLE cases, respectively. However, these antibodies are used to assess secondary Sjögren syndrome in patients with SLE because they are highly associated with Sjögren syndrome with 90% specificity. Urinary biomarkers such as 24-hour urine protein and protein creatinine ratio are conventional urinary biomarkers for lupus nephritis. Anti-U1RNP antibody (anti-U1RNP) is a marker with a low sensitivity and moderate specificity and is mostly used to detect mixed connective tissue disease and systemic sclerosis. Investigations were repeated by the rheumatologist to confirm the diagnosis and rule out other rheumatologic conditions and suspected causes of glomerulonephritis in the patient. This patient tested negative for anti-streptolysin O titer measures antibodies against streptolysin O that is produced by group A *Streptococcus *bacteria as well as proteinase 3-ANCA and myeloperoxidase antibodies that are biomarkers to detect glomerulonephritis or vasculitis.

Although the patient tested positive for anti-U1RNP, according to the diagnostic criteria for mixed connective tissue disease from the Japan Research Committee of the Ministry of Health, Labor, and Welfare for Systemic Autoimmune Diseases, mixed connective tissue disease is diagnosed when a patient meets all the following: at least one common manifestation such as (Raynaud’s phenomenon or Puffy fingers and/or swollen hands), immunological manifestation (positivity for anti-U1RNP), and at least one characteristic organ involvement (pulmonary arterial hypertension, aseptic meningitis or trigeminal neuropathy), or when a patient meets all the following: at least one common manifestation, immunological manifestation, and at least one feature each in two or more from items A (SLE-like manifestations), B (systemic sclerosis-like manifestations), and C (polymyositis/dermatomyositis-like manifestations) in overlapping manifestations. This patient had only the immunological manifestation (positive anti-U1RNP) and did not have systemic sclerosis-like manifestations. Thus, he did not meet the criteria for mixed connective tissue disease.

Our patient was diagnosed as having SLE with positive 5g protein/24 hour, ANA, low C3, anti-U1RNP, anti-Ro, and anti-La antibodies, as well as membranous lupus nephritis, as evident by the results of renal biopsy. The diagnosis of myositis was also made according to the history and evaluation of the patient, the high titer of muscle enzymes CK level, and MRI result. Eventually, the patient was diagnosed with overlap syndrome.

Several published series and reports have discussed SLE-myositis overlap. However, almost all patients (unlike our case) with SLE-myositis overlap were females [7-12,13]. The high prevalence in females is particularly interesting, and several explanations have been suggested. One of the famous explanations is the differences between males and females in the metabolism of sex hormones, in particular, estrogen which has been shown to modulate the interferon-alpha pathway resulting in an increased production of inflammatory cytokines. Another explanation for female predominance is attributed to genetics, most notably the *methyl-CpG-binding protein 2* (*MECP2*) and the *osteopontin *gene. The *MECP2 *gene suppresses the transcription of some other methylated genes. A loss of function mutation in this gene has been recently associated with increased susceptibility to the development of autoimmune diseases, in general, and SLE, in particular. This gene is located on chromosome X, and, interestingly, SLE patients have been found to have an overexpression of methylation-sensitive genes. *Osteopontin *(*SPP1*), besides its role in bone biology, is an enhancer of the pro-inflammatory Th1 cell response, an inhibitor of the Th2 response, and plays an important role in the production of interferon-alpha. *Osteopontin *was found to be overexpressed in biopsies taken from inflamed tissues of SLE patients. Surprisingly, estrogen via a non-classic pathway induces *osteopontin* expression, although this expression was found to decrease with age in females [14].

Regarding the management of this case, glucocorticoids are usually used in the flare-up of SLE and myositis to achieve remission while tapering the dose and replacing it with hydroxychloroquine or an immunosuppressant. Nevertheless, patients with lupus nephritis are treated with an anti-inflammatory immediately, followed by a potent immunosuppressive agent usually for three to six months, and a maintenance phase that lasts for three years [2,4,6].

According to current literature, patients with SLE-myositis overlap are less likely to develop renal complications but much more likely to develop pulmonary diseases, erosive joint diseases, alopecia, and oral ulcers. In our case, the patient had class V membranous lupus nephritis. He was started on methylprednisolone injections for three days, then switched to oral prednisolone for the flare and started on mycophenolate mofetil [15-18]. Because our patient did not improve on the initial doses, the doses were increased until he showed symptomatic improvement.

## Conclusions

We would like to bring to the clinician’s attention the possibility of SLE-myositis overlap syndrome in males with minimal non-specific manifestations that are resistant to symptomatic treatments to approach the diagnosis and avoid treatment delay.

Over the course of three months, the patient presented with only one feature of myositis and one feature of SLE that rarely occur in male patients. During follow-ups, the patient started developing other features of SLE such as malar rash. The patient also had a positive serology, imaging, and biopsy of these two inflammatory conditions simultaneously. The patient started to respond well after a few months of treatment with prednisolone and mycophenolate mofetil.
